# Trends in short-term survival from distant-stage cutaneous melanoma in the United States, 2001-2013 (CONCORD-3)

**DOI:** 10.1093/jncics/pkaa078

**Published:** 2020-09-14

**Authors:** Veronica Di Carlo, Jacques Estève, Christopher Johnson, Fabio Girardi, Hannah K Weir, Reda J Wilson, Pamela Minicozzi, Rosemary D Cress, Charles F Lynch, Karen S Pawlish, Judith R Rees, Michel P Coleman, Claudia Allemani, US CONCORD Working Group, T Freeman, J T George, R M Avila, D K O'Brien, A Holt, L Almon, S Kwong, C Morris, R Rycroft, L Mueller, C E Phillips, H Brown, B Cromartie, A G Schwartz, F Vigneau, G M Levin, B Wohler, R Bayakly, K C Ward, S L Gomez, M McKinley, R Cress, M D Green, K Miyagi, C J Johnson, L P Ruppert, C F Lynch, B Huang, T C Tucker, D Deapen, L Liu, M C Hsieh, X C Wu, M Schwenn, K Stern, S T Gershman, R C Knowlton, G Alverson, T Weaver, S Bushhouse, D B Rogers, J Jackson-Thompson, D Lemons, H J Zimmerman, M Hood, J Roberts-Johnson, J R Rees, B Riddle, K S Pawlish, A Stroup, C Key, C Wiggins, A R Kahn, M J Schymura, S Radhakrishnan, C Rao, L K Giljahn, R M Slocumb, R E Espinoza, F Khan, K G Aird, T Beran, J J Rubertone, S J Slack, J Oh, T A Janes, S M Schwartz, S W Bolick, D M Hurley, M A Whiteside, P Miller-Gianturco, M A Williams, K Herget, C Sweeney, A T Johnson, M B Keitheri Cheteri, P Migliore Santiago, S E Blankenship, S Farley, R Borchers, R Malicki, J R Espinoza, J Grandpre, H K Weir, R Wilson, B K Edwards, A Mariotto

**Affiliations:** 1 Cancer Survival Group, Department of Non-Communicable Disease Epidemiology, London School of Hygiene and Tropical Medicine, London, UK; 2 Université Claude Bernard, Hospices Civils de Lyon, Service de Biostatistique, Lyon Cedex 03, France; 3 Cancer Data Registry of Idaho, Boise, ID, USA; 4 Division of Cancer Prevention and Control, Centers for Disease Control and Prevention, Atlanta, GA, USA; 5 Public Health Institute, Cancer Registry of Greater California, Sacramento, CA, USA; 6 Department of Epidemiology, University of Iowa, Iowa City, IA, USA; 7 New Jersey Department of Health, Trenton, NJ, USA; 8 Department of Epidemiology, Geisel School of Medicine, Dartmouth College, Lebanon, NH, USA

## Abstract

**Background:**

Survival from metastatic cutaneous melanoma is substantially lower than for localized disease. Treatments for metastatic melanoma have been limited, but remarkable clinical improvements have been reported in clinical trials in the last decade. We described the characteristics of US patients diagnosed with cutaneous melanoma during 2001-2013 and assessed trends in short-term survival for distant-stage disease.

**Methods:**

Trends in 1-year net survival were estimated using the Pohar Perme estimator, controlling for background mortality with life tables of all-cause mortality rates by county of residence, single year of age, sex, and race for each year 2001-2013. We fitted a flexible parametric survival model on the log-hazard scale to estimate the effect of race on the hazard of death because of melanoma and estimated 1-year net survival by race.

**Results:**

Only 4.4% of the 425 915 melanomas were diagnosed at a distant stage, cases diagnosed at a distant stage are more commonly men, older patients, and African Americans. Age-standardized, 1-year net survival for distant-stage disease was stable at approximately 43% during 2001-2010. From 2010 onward, survival improved rapidly, reaching 58.9% (95% confidence interval = 56.6% to 61.2%) for patients diagnosed in 2013. Younger patients experienced the largest improvement. Survival for distant-stage disease increased in both Blacks and Whites but was consistently lower in Blacks.

**Conclusions:**

One-year survival for distant-stage melanoma improved during 2001-2013, particularly in younger patients and those diagnosed since 2010. This improvement may be a consequence of the introduction of immune-checkpoint-inhibitors and other targeted treatments for metastatic and unresectable disease. Persistent survival inequalities exist between Blacks and Whites, suggesting differential access to treatment.

The incidence of cutaneous melanoma has been rising in most Caucasian populations during the past 50 years (1). In the United States, the age-standardized incidence rate rose from 8 per 100 000 person-years in 1975 to 25 in 2016 (2). Cutaneous melanoma was the fourth and fifth most common cancer in men and women, respectively, in the United States in 2016, with a total of 82 476 new cases (3).

The third cycle of the CONCORD programme for the global surveillance of cancer survival (CONCORD-3) highlighted increasing trends in age-standardized 5-year net survival from cutaneous melanoma in most countries during 2000-2014; 5-year net survival exceeded 90% for patients diagnosed during 2010-2014 in the United States, Australia, New Zealand, and most Nordic and Western European countries but was below 60% in Ecuador, China, and Taiwan (4). Stage at diagnosis is an important predictor of prognosis, and survival for disease diagnosed at an advanced stage is much lower than for localized disease. If detected at a localized stage (tumor node metastasis [TNM] stage I-II and resectable stage III), cutaneous melanoma can be surgically treated with a favorable outcome. Five-year relative survival for localized melanoma of the skin diagnosed in the last 20 years was higher than 90% in Germany (5), Denmark (6), Estonia (7), Sweden (8), and the United States (9).

Until about 2010, when advanced disease (TNM stage III unresectable melanoma and stage IV disease) was mainly treated with chemotherapy (eg, dacarbazine) and cytokines (eg, interleukin-2), the prognosis for metastatic melanoma was generally poor, with survival as low as 16% at 5 years after diagnosis for patients diagnosed in the United States (9,10). In recent years, major improvements in treatment, involving the use of targeted therapies and immunotherapy, have led to unprecedented clinical benefit. Ipilimumab, the first immunotherapy, and vemurafenib, the first targeted treatment for metastatic and unresectable melanoma, were approved by the US Food and Drug Administration (FDA) in 2011.

The aim of this study is to describe the characteristics of patients diagnosed with cutaneous melanoma during 2001-2013 using data provided by 34 US population-based cancer registries included in CONCORD-3 and to assess trends in short-term (1-year) survival for distant-stage disease.

## Methods

CONCORD-3 obtained anonymized, individual tumor records from 322 population-based cancer registries in 71 countries worldwide, for patients who had been diagnosed with one of 18 common cancers, including melanoma, during 2000-2014 and followed-up to December 31, 2014. Data acquisition, ethical approval, and data quality control for the CONCORD programme have been described elsewhere (4). Cancer registries submitted records on all patients diagnosed with a melanoma, defined by morphology codes in the range 8720-8790 in the International Classification of Diseases for Oncology, third revision (ICD-O-3) (11). We restricted survival analysis to malignant melanoma (ICD-O-3 behavior code 3) arising in the skin (ICD-O-3 topography codes C44.0-C44.9), including the skin of the labia majora (C51.0), vulva (C51.9), penis (C60.9), and scrotum (C63.2).

Records with incomplete data or for tumors that were benign, in situ, of uncertain behavior, metastatic from another organ, or unknown if primary or metastatic, or on patients with age outside the range 15-99 years, were considered ineligible for analysis.

We excluded tumors registered only from a death certificate or discovered at autopsy, because their duration of survival was unknown, as well as records for which the vital status or sex was unknown and those with an invalid date or sequence of dates.

We included in analysis only primary, invasive, malignant cutaneous melanoma. If two or more invasive primary malignant melanomas were detected in the same person but with different dates of diagnosis, the record with the earliest date of diagnosis was retained. Registry datasets in which 15.0% or more of patients were lost to follow-up were excluded from the survival analyses.

Patients diagnosed in 2014 were included in CONCORD-3 but were not included in this study, because a full year of follow-up was not available by the study closure date (December 31, 2014). To assess trends in survival for the same registries, we retained only registries that submitted data on patients diagnosed up to and including 2013, with follow-up to December 31, 2014.

The CONCORD protocol required information on stage of disease at the time of diagnosis for patients diagnosed from 2001 onward, because the completeness of data on stage in many countries and United States was known to be much lower before 2001.

Stage was categorized as localized, regional, and distant according to the Surveillance, Epidemiology, and End Results Summary Stage 2000 classification (12). “Distant stage” includes melanoma with distant lymph node involvement, metastatic skin lesions, further contiguous extension, or metastasis to other organs. Age at diagnosis was grouped into 15-44 years, 45-54 years, 55-64 years, 65-74 years, and 75-99 years. Race was categorized as White, Black, and other race or ethnicities (Asian or Pacific Islander; American Indian or Alaska Native; other, unspecified or unknown race).

Melanomas were defined by morphology (ICD-O-3 8720–8790). We selected melanomas of the skin on the basis of topographic codes C44.0-C44.9 (skin), C51.0 (including the skin of the labia majora), C51.9 (vulva), C60.9 (penis), or C63.2 (scrotum). Melanomas were further categorized by anatomic subsite as arising in the skin of the head and neck (C44.0-C44.4), the trunk (C44.5), the limbs (C44.6-C44.7), or the genital organs (C51.0, C51.9, C60.9, C63.2), as lesions overlapping 2 of those categories, or of the skin with anatomic location not otherwise specified (C44.8-C44.9). Histological subtypes were grouped according to the first revision of ICD-O-3 (11) as malignant melanoma, not otherwise specified (NOS, 8720), superficial spreading (8743), lentigo maligna (8742), nodular (8721), acral (8744), and all other morphologies (8722-8723, 8726-8727, 8730, 8740-8741, 8743, 8745-8746, 8750, 8760-8761, 8770-8774, 8780, 8790).

We explored the distribution of stage at diagnosis by sex, age, race, topography, and morphology. Survival analyses were restricted to patients diagnosed with distant-stage melanoma. One-year net survival for patients diagnosed in each of the 13 years from 2001 to 2013 was estimated with the non-parametric Pohar Perme estimator (13) using the STATA (14) command *stns* (15). Net survival is the cumulative probability that cancer patients survive their cancer up to a given time since diagnosis (eg, 1 year) after correcting for other causes of death (background mortality). To control for background mortality, which varies by geographical area, demographic characteristics, and over time, we used life tables of all-cause mortality in the general population by single year of age, sex, single calendar year, race (Blacks, Whites, and others) and county within each state. These life tables were kindly provided by the National Cancer Institute (16).

We estimated trends in 1-year net survival for 5 age groups. We then obtained age-standardized estimates for all ages combined using the second of the 3 sets of International Cancer Survival Standard weights (0.28, 0.17, 0.21, 0.20, and 0.14) designed for cancers with broadly constant incidence by age (17). Survival was estimated for men and women, and for both sexes combined.

We fitted a flexible parametric survival model on the log-hazard scale to estimate the effect of race on the hazard of death because of distant-stage melanoma; excess mortality and net survival by race were also estimated (18), with race as a categorical variable. Restricted cubic splines for the effect of age at diagnosis (3 degrees of freedom) and year of diagnosis (4 degrees of freedom) were included with the command *rcsgen* (19), including time-dependent effects.

## Results

The CONCORD database included individual records for 1 040 814 adults (15-99 years) diagnosed with a primary, malignant cutaneous melanoma in 41 state-wide cancer registries in the United States covering a total population of 257 million people (80.2% of the US population). Data quality was generally high. The proportion of patients excluded for incomplete dates or for other reasons ranged from 0.0% to 4.4% (Table 1). Overall, 36.0% of patients were diagnosed with an in situ tumor.

**Table 1.** Data quality indicators: patients diagnosed with malignant melanoma of the skin during 2000-2014 in the United States

US registries	Calendar period	No. of patients submitted	Ineligible, %^a^			No. of eligible patients	Excluded, %^b^		No. of patients included	Data quality indicators, %^c^	
			Incomplete dates	In situ	Other		DCO	Other		Lost to follow-up	Censored
All US registries	2000-2014	1 040 814	0.6	36.0	2.6	632 861	0.5	0.0	629 816	2.6	0.1
Alabama	2000-2014	23 564	0.9	41.3	2.3	13 084	0.6	0.0	13 012	0.0	0.0
Alaska	2000-2013	1533	4.4	30.6	3.5	944	0.4	0.0	940	0.0	0.0
Arkansas	2000-2011	7592	0.3	31.9	3.3	4897	0.3	0.0	4879	0.0	0.0
California	2000-2011	127 043	1.1	36.9	2.3	75 851	0.2	0.0	75 712	0.0	0.0
Colorado	2000-2013	21 135	0.3	33.1	3.1	13 427	0.7	0.0	13 338	0.0	0.0
Connecticut	2000-2014	21 602	0.4	40.9	2.2	12 211	0.2	0.0	12 185	5.5	0.0
Delaware	2000-2014	6283	0.2	44.0	1.4	3413	0.2	0.0	3406	0.0	0.0
Florida	2000-2013	89 847	0.1	35.4	2.7	55 590	0.7	0.1	55 134	0.0	0.0
Georgia	2000-2014	43 981	0.0	35.6	2.0	27 451	0.4	0.0	27 350	0.0	0.0
Hawaii	2000-2014	5753	0.3	33.7	1.5	3710	0.2	0.0	3704	7.5	0.0
Idaho	2000-2014	9032	0.6	40.8	2.2	5095	0.7	0.0	5059	0.0	0.0
Indiana	2000-2014	25 599	0.6	32.3	3.3	16 347	0.5	0.0	16 269	0.0	0.0
Iowa	2000-2014	15 612	0.6	32.6	3.7	9846	0.2	0.0	9822	2.8	0.0
Kentucky	2000-2014	23 097	0.0	33.3	2.8	14 764	0.2	0.0	14 729	6.4	0.0
Louisiana	2000-2014	15 105	0.5	37.1	2.8	9000	0.2	0.0	8982	6.4	0.1
Maine	2000-2013	7860	0.3	38.4	3.0	4581	0.3	0.0	4565	0.0	0.0
Maryland	2000-2014	29 516	0.4	40.2	1.8	16 981	0.6	0.1	16 868	0.0	0.0
Massachusetts	2000-2009	23 194	0.0	34.5	3.0	14 483	0.4	0.0	14 420	0.0	0.0
Michigan	2000-2013	41 986	0.2	36.5	2.5	25 505	0.6	0.0	25 335	0.0	0.0
Minnesota	2000-2013	27 449	0.0	38.1	1.9	16 472	0.3	0.0	16 421	0.0	0.0
Mississippi	2002-2014	9214	0.8	31.6	2.8	5968	0.6	0.0	5931	0.0	0.0
Montana	2000-2014	5595	0.6	37.8	2.9	3289	0.5	0.0	3272	0.0	0.0
Nebraska	2000-2014	7894	0.6	33.4	3.5	4930	0.5	0.0	4906	0.0	0.0
New Hampshire	2000-2014	9727	0.1	40.3	2.3	5575	0.3	0.0	5560	0.0	0.0
New Jersey	2000-2014	49568	0.8	42.7	1.9	27 024	0.4	0.0	26 910	48.2	0.0
New Mexico	2000-2014	8720	0.0	40.1	2.2	5030	0.6	0.0	5000	8.7	0.4
North Carolina	2000-2014	47 654	0.0	39.5	2.4	27 727	0.4	0.0	27 602	0.0	0.0
Ohio	2000-2014	54 382	0.1	35.7	3.0	33 292	0.6	0.0	33 079	0.0	0.0
Oklahoma	2000-2010	9135	0.4	24.8	3.9	6479	1.1	0.0	6407	0.0	0.0
Oregon	2000-2013	24 301	0.1	40.9	2.6	13 703	0.5	0.0	13 637	0.0	0.0
Pennsylvania	2000-2014	62 912	2.4	32.9	2.7	39 052	0.4	0.0	38 904	0.0	0.0
Rhode Island	2000-2014	6363	0.4	39.0	2.4	3703	0.4	0.0	3688	0.0	0.0
South Carolina	2000-2014	24 940	0.0	40.8	1.8	14 309	0.5	0.0	14 230	0.0	0.0
Tennessee	2000-2011	19 264	0.5	28.5	3.3	13 047	0.3	0.0	13 003	0.0	0.0
Texas	2000-2013	59 374	0.9	28.4	3.5	39 862	0.8	0.0	39 555	0.0	0.0
Utah	2000-2014	14 946	0.1	38.2	2.1	8893	0.1	0.0	8885	0.0	0.2
Vermont	2000-2013	4537	0.1	38.8	1.9	2688	0.3	0.0	2679	0.0	0.0
Washington	2000-2008	22 317	0.8	39.2	2.2	12 876	0.2	0.0	12 843	0.0	0.0
West Virginia	2000-2014	8894	1.3	31.1	3.4	5707	0.4	0.0	5682	0.0	0.0
Wisconsin	2000-2013	21 636	0.9	28.4	3.6	14 507	1.0	0.0	14 366	0.0	0.0
Wyoming	2000-2013	2658	0.2	38.6	2.9	1548	0.1	0.0	1547	0.0	0.1

a Incomplete dates: records in which the year of birth is unknown, the month and/or year of diagnosis is unknown, or the year of last known vital status is unknown. Other: records with incomplete data or for tumors that are benign (behavior code 0), of uncertain behavior (1), metastatic from another organ (6), or unknown if primary or metastatic (9); or for patients with age outside the range of 15-99 years. DCO = Tumours registered only from a death certificate.

b Other: vital status or sex unknown; invalid date or sequence of dates.

c Censored: patients whose last known vital status is “alive” and who were censored within 5 years of diagnosis or, if diagnosed in 2010 or later, before December 31, 2014.

Of the 632 861 patients eligible for inclusion in survival analyses, we excluded 3045 (0.5%) because the cancer was registered only from a death certificate or discovered at autopsy; survival time for these patients is unknown. Only 2.7% of the remaining 629 816 patients were lost to follow-up or censored within 5 years from diagnosis, but this proportion was much lower among patients with distant-stage disease (0.3%). The diagnosis was histologically confirmed in 99.3% of tumors (data not shown).

New Jersey was excluded because of the high proportion of patients lost to follow-up (48.2%). A further 118 239 patients were excluded from 6 state-wide registries (Arkansas, California, Massachusetts, Oklahoma, Tennessee, and Washington), because data were not available for patients diagnosed up to and including 2013. Finally, we explored the distribution of 425 915 patients by sex, age, race, topography, morphology, and stage at diagnosis.

Most patients diagnosed during 2001-2013 were men (56.8%), and they were generally older than women (median age at diagnosis = 64 vs 57 years, respectively). Only 0.6% of patients were Black (Table 2). Data on stage at diagnosis were available for 386 885 (90.8%) patients.

**Table 2.** Adults (15-99 years) diagnosed with primary malignant melanoma of the skin during 2001-2013 in 34 US registries: distribution by sex, age at diagnosis, race, anatomic location, morphology, and SEER Summary Stage 2000^a^

Patient and tumor characteristics	Localized	Regional	Distant	Unknown	Total
	No. (%)	No. (%)	No. (%)	No. (%)	No. (%)
Sex					
Male	182 150 (75.3)	24 747 (10.2)	12 443 (5.1)	22 470 (9.4)	241 810 (56.8)
Female	146 022 (79.3)	15 365 (8.3)	6158 (3.3)	16 560 (9.1)	184 105 (43.2)
Age group, y					
15-44	61 321 (79.7)	7039 (9.1)	2074 (2.7)	6510 (8.5)	76 944 (18.1)
45-54	58 041 (78.2)	6857 (9.2)	2942 (4.0)	6386 (8.6)	74 226 (17.4)
55-64	69 434 (77.4)	8296 (9.2)	4131 (4.6)	7848 (8.8)	89 709 (21.1)
65-74	66 251 (76.8)	7739 (9.0)	4204 (4.9)	8116 (9.3)	86 310 (20.3)
75-99	73 125 (74.1)	10 181 (10.3)	5250 (5.3)	10 170 (10.3)	98 726 (23.2)
Race					
White	315 166 (77.3)	39 200 (9.6)	18 052 (4.4)	35 550 (8.7)	407 968 (95.8)
Black	1286 (51.8)	500 (20.1)	363 (14.6)	333 (13.5)	2482 (0.6)
Other	11 720 (75.8)	412 (2.7)	186 (1.2)	3147 (20.3)	15 465 (3.6)
Anatomic location					
Head and neck	67 980 (77.6)	9140 (10.4)	2036 (2.3)	8405 (9.7)	87 561 (20.6)
Trunk	111 247 (81.3)	12 071 (8.8)	2817 (2.1)	10 754 (7.8)	136 889 (32.1)
Limbs	146 001 (81.5)	16 259 (9.1)	3314 (1.9)	13 561 (7.5)	179 135 (41.1)
Overlapping region or NOS	2014 (9.7)	2297 (11.0)	10 321 (49.6)	6191 (29.7)	20 823 (4.9)
Skin of genital organs	930 (61.7)	345 (22.9)	113 (7.5)	119 (7.9)	1507 (0.4)
Morphology					
Malignant melanoma, NOS	156 892 (1.8)	17 992 (8.2)	14 538 (6.7)	29 031 (13.3)	225 635 (51.9)
Superficial spreading	115 022 (89.0)	7906 (6.1)	1077 (0.8)	5285 (4.1)	129 782 (29.8)
Lentigo maligna	23 590 (88.0)	808 (3.0)	162 (0.6)	2258 (8.4)	27 163 (6.2)
Nodular	19 161 (62.1)	8963 (29.1)	1653 (5.4)	1064 (3.4)	31 329 (7.2)
Acral lentiginous	2990 (68.2)	1017 (23.2)	189 (4.3)	186 (4.3)	4428 (1.0)
Others	10 517 (65.2)	3426 (21.2)	982 (6.1)	1206 (7.5)	16 518 (3.8)
Total	328 172 (77.1)	40 112 (9.4)	18 601 (4.4)	39 030 (9.1)	425 915 (100.0)

a NOS = not otherwise specified; SEER = Surveillance, Epidemiology, and End Results.

A majority of patients (77.1%) were diagnosed with localized disease. This proportion was stable over time (76.4%-79.8%, data not shown) and slightly higher in women (79.3% vs 75.3%) and in younger patients (79.7% vs 74.1% in patients aged 15-44 years and 75-99 years, respectively). Of melanomas, 4.4% were diagnosed at a distant stage, with a slightly higher proportion in men than women (4.6% vs 2.8% respectively, in 2001; 6.2% vs 4.5% in 2013, data not shown). There were 14.6% of Blacks diagnosed with distant-stage disease compared with only 4.4% in Whites and 1.2% in the “other race” category. Patients with distant-stage melanoma were generally older (median age = 65 years) than those diagnosed with localized (61 years) or regional (62 years) disease (data not shown).

Melanomas arose mostly on the skin of the limbs (42.1%), the trunk (32.1%), and the head and neck (20.6%) and were diagnosed at a distant stage in 2.0% of those cases (Table 2). Melanomas arising in overlapping or unspecified locations accounted for only 4.9% of all cases, but about one-half of these (49.6%) were diagnosed at an advanced stage. The proportion of melanomas registered with an unspecified morphology was 51.9%, followed by superficial spreading (29.8%) and nodular melanoma (7.2%). Distant-stage melanomas represented less than 1% of the superficial spreading and lentigo maligna morphologies (0.8% and 0.6%, respectively), but up to 6.7% of those classified as malignant melanoma NOS.

**Figure 1.**
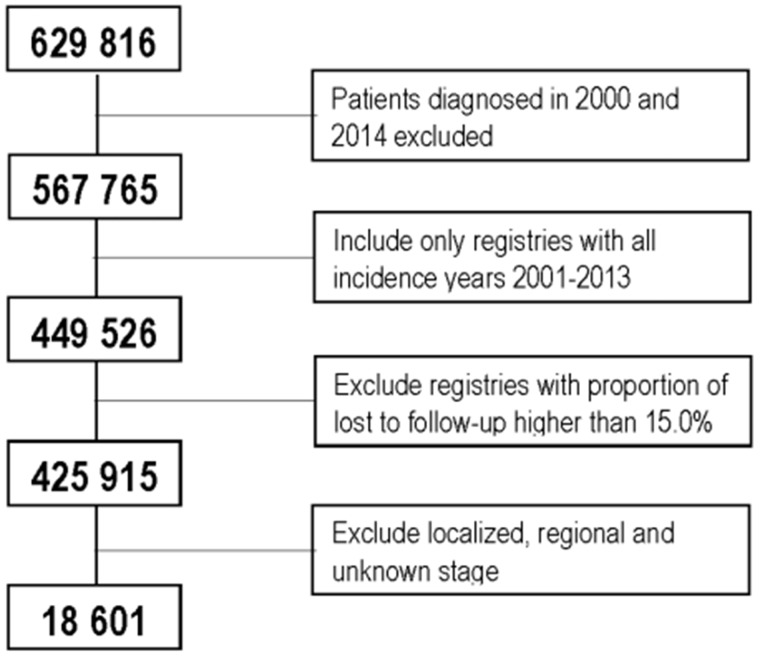
Patients included in survival analysis.

We restricted survival analysis to 18 601 patients diagnosed with distant-stage disease (Figure 1). In 2001, age-standardized 1-year net survival was 42.8% (95% confidence interval [CI] = 39.3% to 46.3%) and remained stable until 2010 (Table 3). Survival improved rapidly from 2010 onward, reaching 58.9% (95% CI = 56.6% to 61.2%) for patients diagnosed in 2013. The trend was similar for men and women, although survival was slightly but consistently higher in women (Table 3).

**Table 3.** Number of patients at risk together with age-standardized and age-specific 1-year net survival for patients diagnosed with distant-stage cutaneous melanoma during 2001-2013 in 34 US registries overall, by sex, and by age at diagnosis^a^

Calendar year	US registries		Sex				Age, y									
			Men		Women		15-44		45-54		55-64		65-74		75-99	
	No.	NS, % (95% CI)	No.	NS, % (95% CI)	No.	NS, % (95% CI)	No.	NS, % (95% CI)	No.	NS, % (95% CI)	No.	NS, % (95% CI)	No.	NS, % (95% CI)	No.	NS, % (95% CI)
2001	921	42.8 (39.3 to 46.3)	626	39.9 (35.7 to 44.1)	295	48.7 (42.5 to 54.9)	132	44.4 (35.9 to 52.8)	178	45.7 (38.4 to 53.1)	169	50.2 (42.6 to 57.8)	198	32.7 (26.1 to 39.4)	244	39.7 (33.0 to 46.3)
2002	1009	38.5 (35.2 to 41.7)	673	36.8 (32.9 to 40.7)	336	41.6 (35.9 to 47.2)	162	46.4 (38.7 to 54.0)	186	34.0 (27.2 to 40.8)	198	37.3 (30.5 to 44.0)	208	36.1 (29.5 to 42.7)	255	33.2 (27.1 to 39.3)
2003	1070	44.1 (40.7 to 47.4)	733	42.3 (38.3 to 46.3)	337	48.0 (42.1 to 53.9)	133	49.7 (41.3 to 58.2)	185	44.5 (37.4 to 51.7)	230	45.3 (38.8 to 51.7)	244	42.8 (36.5 to 49.2)	278	32.3 (26.5 to 38.1)
2004	1226	42.9 (39.8 to 46.0)	807	40.0 (36.2 to 43.9)	419	48.6 (43.4 to 53.8)	163	46.7 (39.1 to 54.3)	207	38.8 (32.2 to 45.4)	250	42.4 (36.3 to 48.6)	256	42.9 (36.7 to 49.1)	350	40.8 (35.2 to 46.3)
2005	1244	42.8 (39.6 to 46.0)	855	42.5 (38.5 to 46.4)	389	43.2 (37.8 to 48.7)	137	43.9 (35.6 to 52.1)	195	44.3 (37.3 to 51.3)	266	45.4 (39.3 to 51.4)	288	40.5 (34.7 to 46.2)	358	38.5 (33.0 to 43.9)
2006	1359	45.6 (42.5 to 48.7)	879	44.0 (40.2 to 47.8)	480	48.5 (43.4 to 53.7)	146	51.5 (43.4 to 59.5)	232	47.6 (41.2 to 54.0)	312	44.4 (38.8 to 49.9)	297	41.7 (36.0 to 47.4)	372	38.7 (33.4 to 44.0)
2007	1319	44.5 (41.3 to 47.7)	855	44.2 (40.1 to 48.2)	464	45.6 (40.3 to 50.8)	130	45.5 (37.0 to 54.0)	209	43.7 (37.0 to 50.5)	281	45.3 (39.4 to 51.1)	317	48.4 (42.8 to 54.1)	382	37 (31.8 to 42.1)
2008	1381	42.8 (39.7 to 45.9)	935	41.1 (37.2 to 45.0)	446	46.6 (41.5 to 51.8)	142	43 (34.9 to 51.1)	225	47.2 (40.7 to 53.7)	336	40.3 (35.0 to 45.5)	290	45.2 (39.4 to 51.0)	388	37.2 (32.1 to 42.3)
2009	1486	42.0 (39.1 to 45.0)	988	40.5 (36.8 to 44.1)	498	45 (40.0 to 49.9)	159	44.7 (37.0 to 52.4)	230	38.9 (32.6 to 45.2)	346	43.2 (37.9 to 48.4)	341	43.8 (38.4 to 49.2)	410	36.2 (31.3 to 41.2)
2010	1678	45.7 (43.0 to 48.3)	1151	44.5 (41.2 to 47.8)	527	47.9 (43.3 to 52.5)	207	57.1 (50.4 to 63.8)	277	46.1 (40.2 to 51.9)	385	41.4 (36.5 to 46.4)	366	41.4 (36.3 to 46.5)	443	34.9 (30.2 to 39.6)
2011	1725	51.9 (49.2 to 54.6)	1168	49.0 (45.4 to 52.6)	557	56.8 (52.5 to 61.1)	168	66.1 (58.9 to 73.2)	265	51.7 (45.7 to 57.8)	430	45.8 (41.1 to 50.5)	388	47.4 (42.4 to 52.5)	474	39.3 (34.6 to 44.0)
2012	2012	56.7 (54.3 to 59.2)	1355	54.6 (51.4 to 57.7)	657	60.3 (56.4 to 64.1)	226	70.3 (64.4 to 76.3)	297	58.2 (52.5 to 63.8)	485	51.0 (46.5 to 55.5)	486	51.1 (46.6 to 55.7)	518	44.5 (39.9 to 49.1)
2013	2171	58.9 (56.6 to 61.2)	1418	57.4 (54.4 to 60.5)	753	61.4 (57.7 to 65.1)	251	67.8 (62.0 to 73.6)	349	62.7 (57.6 to 67.8)	484	56.1 (51.6 to 60.6)	541	56.7 (52.4 to 60.9)	546	43.9 (39.4 to 48.3)

a CI = confidence interval; NS = net survival.

**Figure 2.**
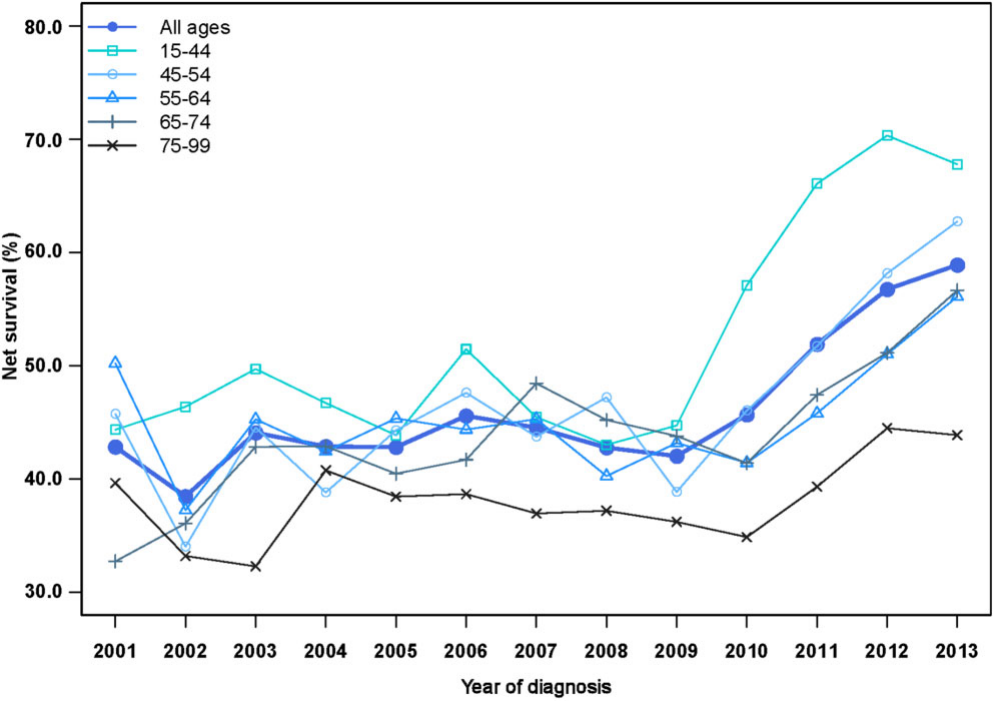
Trends in age-specific 1-year net survival (%) for patients diagnosed with distant-stage cutaneous melanoma during 2001-2013 in the United States.

One-year net survival increased for all ages (Figure 2; Table 3). The youngest patients (15-44 years) experienced the largest absolute improvement, particularly from 2010, increasing from 44.4% (95% CI = 35.9% to 52.8%) in 2001 to 67.8% (95% CI = 62.0% to 73.6%) in 2013. For patients aged 45-54 years, 1-year survival increased from 45.7% (95% CI = 38.4% to 53.1%) in 2001 to 62.7% (95% CI = 57.6% to 67.8%) in 2013. We observed similar trends in patients aged 55-64 years and 65-74 years starting from 2011; both survival curves reached 56% (56.1%, 95% CI = 51.6% to 60.6%; and 56.7%, 95% CI = 52.4% to 60.9%, respectively) in 2013. One-year survival for patients aged 75 years or older remained at 44.5% (95% CI = 39.9% to 49.1%) or lower throughout the period 2001-2013.

**Figure 3.**
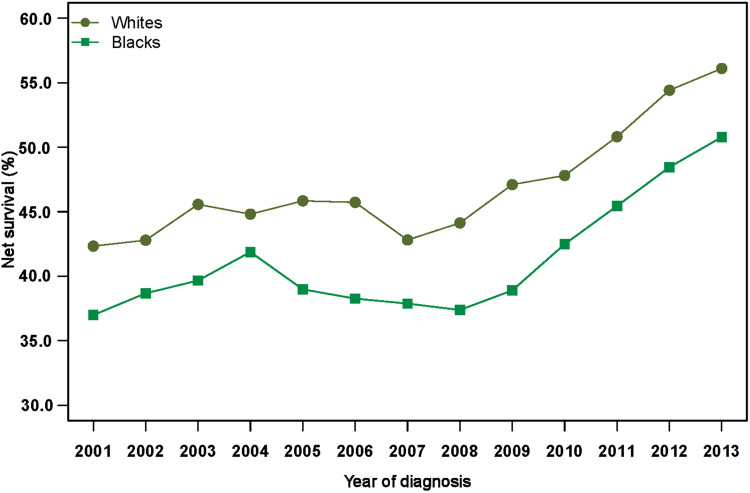
Trends in age-standardized 1-year net survival (%) for patients diagnosed with distant-stage cutaneous melanoma during 2001-2013 in the United States, by race.

Age-standardized 1-year net survival increased for both Whites and Blacks with distant-stage melanoma (Figure 3). Survival for Whites increased from 42.3% (95% CI = 39.9% to 44.8%) in 2001 to 56.1% (95% CI = 54.6% to 57.6%) in 2013. Among Blacks, 1-year survival improved from 37.0% (95% CI = 32.0% to 42.7%) to 50.7% (95% CI = 46.3% to 55.7%) over the same period. The excess hazard of death because of melanoma within 1 year of diagnosis was 13% higher in Blacks than Whites (excess hazard ratio = 1.13, 95% CI = 1.00 to 1.27; data not shown).

## Discussion

This study includes data from 34 state-wide cancer registries, covering 56.9% of the US population and is the largest population-based analysis to date of trends in 1-year survival for distant-stage cutaneous melanoma. It shows a dramatic improvement in survival, particularly between 2010 and 2013.

The proportion of melanomas diagnosed at a distant stage remained stable over time (4%-5%) and was slightly lower in women than men. Sex inequalities in stage at diagnosis are well known (20–22); they are commonly attributed to differences in health-seeking behavior (23). Traditionally, women tend to visit their health-care provider and perform skin checks more frequently than men; this can translate to a higher proportion of women being diagnosed with localized disease.

Blacks were more likely to be diagnosed with distant-stage melanoma than Whites. The perception among African Americans that melanoma risk is low is considered a major cause for delayed diagnosis (24,25). Consistent with previous studies (26–29), patients diagnosed at a distant stage were generally older.

One-year net survival improved noticeably for men and women and in both Blacks and Whites. This improvement may reflect the recent introduction of new treatments for metastatic and unresectable disease.

The first immune checkpoint inhibitor approved by the FDA, ipilimumab (30), in March 2011 showed 1-year overall survival for patients diagnosed with metastatic melanoma in a phase III randomized clinical trial as high as 45.6% compared with less than 30% (25.3%) for patients treated with standard therapy (31).

Vemurafenib, the first licensed targeted treatment for patients with metastatic disease and the BRAF V600E mutation, was also shown to increase short-term survival. A phase III randomized trial of 675 patients diagnosed with metastatic melanoma showed an overall 6-month survival of 84% (95% CI = 78% to 89%) in those treated with vemurafenib compared with 64% (95% CI = 56% to 73%) in those treated with dacarbazine (32). The FDA approved the drug on this evidence in August 2011 (33).

Our study has shown a substantial improvement in short-term survival since 2010-2011 for patients diagnosed with distant-stage melanoma of the skin, particularly for younger patients. Most of the improvement occurred from 2010, one year before FDA approval of the new lines of treatment. Some of these patients may have been recruited to clinical trials, which started well before 2010 (31,34–36). Additionally, they may have received the newer treatments through the FDA expanded access programs (37), which provide access to investigational drugs before their official approval to patients with life-threatening conditions who cannot be enrolled in clinical trials.

Data on whether the patients were recruited to a clinical trial or received systemic therapy for compassionate use were not available to us to explore these hypotheses. However, a population-based study of the impact of targeted and immune-based therapies for metastatic or unresectable melanoma in Ontario found that about 5% of patients were already being treated with the new therapies in 2007; this percentage increased to more than 82% by 2015 (38). That study confirmed the use of immunotherapy well before the approval of ipilimumab by Health Canada in 2012 and highlighted its widespread use in recent years. A similar study in the United States showed that the use of immunotherapy in patients younger than 65 years improved rapidly after 2010, from 8-12% during 2004-2010 to 30% in 2014 (39).

Patients aged 75 years or older with distant-stage disease experienced considerably less improvement in short-term survival. This may be due to less frequent use of the newer therapies. A recent study designed to identify factors associated with the treatment of metastatic melanoma in the United States (40) found that older patients were less likely to receive ipilimumab or to be tested for the BRAF mutation. This may have resulted from concerns about how they would tolerate the new treatments. Previous studies on solid tumors have shown that age can act as a barrier to receipt of optimal treatment because of a higher prevalence of comorbidity or absence of data on treatment efficacy from clinical trials and more frequent adverse effects (41,42). A US study showed that only 46% of patients aged 80 years or older received imatinib, a highly effective treatment for chronic myeloid leukaemia, compared with 89.7% of those aged 20-59 years (43).

The CONCORD-3 study protocol did not require detailed information on specific types of treatment, so it was not possible to estimate the proportion of patients who received immune-checkpoint inhibitors or targeted treatments. Data on socio-economic status and type of health insurance were not collected. That information might have helped to explain the disparities in the stage distribution and stage-specific survival by age and race. An analysis of 61 650 melanoma patients aged 18-64 years diagnosed in the United States during 2007-2012 estimated that the proportion of patients with metastatic disease ranged from only 3.7% in the non-Medicaid insurance group to 15.5% among Medicaid and 10.7% among uninsured patients (44). A recent systematic review of the cost-effectiveness of immune-checkpoint inhibitors in the United States estimated that the individual cost of treatment for metastatic melanoma ranged from US$152 000 to US$303 000 for a patient with a median survival time (45). The cost of targeted therapies for metastatic melanoma with the BRAF V600E mutation was estimated at between US$149 000 and US$319 000 (46). Recent analyses have shown that patients were less likely to receive immunotherapy if they had no insurance or only Medicaid coverage, received a lower income, or received care at a community practice rather than an academic center (39,47,48). Such differences in access to treatment may partly explain the racial disparities in the recent trends in short-term survival reported in this study.

One-year net survival was consistently lower in Blacks than Whites. Survival was not estimated for other races. The proportion of patients lost to follow-up, including those whose deaths are missed by the cancer registries, is generally higher among Asians or Pacific Islanders than Whites and Blacks (49,50). Incomplete follow-up among Asians or Pacific Islanders and other minority groups may lead to overestimation of survival and biased comparisons.

Several studies have shown a survival disadvantage for Blacks diagnosed with melanoma in the United States. A study of more than 260 000 people diagnosed during 1988-2011 estimated an absolute gap of almost 20% (89% vs 70%) between Blacks and Whites in 5-year relative survival for all stages combined (26). Among Whites and Blacks of non-Hispanic origin, the difference in 5-year overall survival was almost 30% (82% vs 53%) during 1982-2011 (27).

Racial disparities in survival from melanoma have commonly been ascribed to a less favorable stage distribution of Black patients (26,51–53). However, we have shown that the proportion of distant-stage melanoma was higher among Blacks than Whites, and 1-year survival for distant-stage melanoma was consistently lower among Blacks than among Whites. This gap in survival suggests racial differences in treatment and access to care.

Despite the exclusion of about 2500 patients registered with a distant-stage melanoma in cancer registries for which incidence data were not complete for 2001-2013, we were nevertheless able to include 18 601 patients: this, to our knowledge, is the largest population-based analysis of trends in 1-year net survival for distant-stage disease.

In conclusion, to our knowledge, this is the first population-based study to show a recent improvement in short-term survival from distant-stage cutaneous melanoma in the United States. This may be due to the availability of new and more effective therapies for the treatment of metastatic or unresectable disease. The dramatic improvement since 2010 in short-term survival for melanoma of the skin diagnosed at the metastatic or unresectable stage is important, because for most other solid tumors, survival for metastatic disease has not changed for several decades (54–56). More detailed population-based studies would help evaluate access to novel treatments and their longer term survival benefit for patients diagnosed with distant-stage melanoma.

## Funding

This project was supported by the American Cancer Society, Centers for Disease Control and Prevention, Swiss Re, Swiss Cancer Research Foundation, Swiss Cancer League, Institut National du Cancer, La Ligue Contre le Cancer, Rossy Family Foundation, US National Cancer Institute, and the Susan G. Komen Foundation.

## Notes

**Role of the funder:** The funders had no role in the design of the study; the collection, analysis, and interpretation of the data; the writing of the manuscript; and the decision to submit the manuscript for publication.

**Disclosures:** The authors have no conflicts of interest to declare.

**Role of the authors:** Conceptualization: VDC, CA; Data: all US authors in participating cancer registries; Methodology: VDC, CA; Formal analysis: VDC; Visualization: VDC, CA; Supervision: CA, MPC; Validation: all authors; Writing—original draft: VDC, CA, MPC; Writing—review and editing: all authors; Funding acquisition: CA, MPC.

**Disclaimer:** The findings and conclusions in this article are those of the authors and do not necessarily represent the official position of the Centers for Disease Control and Prevention.

## Data availability statement

The data underlying this article cannot be shared because they are personal data, provided in anonymized form by participating US cancer registries to the CONCORD programme under relevant ethical and statutory approvals in the United States and the United Kingdom, to protect the privacy of individuals. Requests for data should be addressed to the registry or registries concerned.
